# Therapeutics

**Published:** 1893-12

**Authors:** 

**Affiliations:** Demonstrator of Physiology and Lecturer on the History of Medicine in the Medical Department of the University of Texas, etc.


### DEPARTMENT OF THERAPEUTICS.

EDITED BY DAVID CERNA, M. D., PH. D.,
Demonstrator of Physiology and Lecturer on the History of Medicine in the Medical Department of the University of Texas, etc.

AMMONIUM CHLORIDE IN CYSTITIS.—Ammonium is said by G. Corrie, *Virginia Medical Monthly*, No. 6, 1893, to be superior to all other remedies in relieving cystitis,—in preparing cases for operation, and in palliating those unsuitable for operation. The author has administered this salt, with very satisfactory results in every instance, in cystitis dependent upon stone in the bladder in cystic irritation from uterine disease or menstrual disorders, in cystic and renal sequelæ of influenza, etc. He prescribes, as a rule, a No. 1 capsuleful of pulverized ammonium chloride, to be taken three to four times in the twenty-four hours (preferably when the stomach is rather empty), each dose to be immediately followed by a glass of cold water. He advises to fill the capsules only when needed, as the salt will dissolve the gelatin in a short time.—*American Medico-Surgical Bulletin*, November, 1893.

CHLOROFORM AS A TÆNICIDE.—Stephen (*La Semaine* Medicale, No. 54, 1893), again calls attention to the great value of chloroform as a tænicide. The author obtained the expulsion of the worm (*T. solium* and *T. mediocanellata*), even when all other remedies had completely failed. He employed the following prescription:

℞ Chloroform......... 4 grammes (1 fluid drachm);
Syrup.............30 grammes (6 fluid drachms).

To be taken in four parts, at 7, 9 and 11 o'clock in the morning, and at 1 o'clock in the afternoon. The patient is advised to take, besides, 30 grammes (1 fluid ounce) of castor oil, at noon. It is stated that the chloroform was always well tolerated.

DEATH FROM THE USE OF PENTAL.—Lick (*Deut. Medical Woch.—Rev. Gen. de Clin. et de Therap.*, September, 1893), reports two fatal cases of poisoning by pental. A young girl, whose necrosed finger it was necessary to amputate, inhaled ten grammes of pental from a sponge inhaler. During the anæsthesia dyspnœa, cyanosis and cardiac failure suddenly developed. Artificial respiration and tracheotomy were of no avail, so that death soon followed. The second case was a girl, aged 18, anæsthetized by pental for the injection of iodoform into the hip. She had been previously anæsthetized by chloroform and bromide of ethyl. On removing the inhaler ten minutes after the inhalation had begun, dyspnœa, spasm of the masseters, sterterous breathing and cyanosis developed. Injections of ether, artificial respiration, injections of salt water, and tracheotomy were futile; the patient perished. In these cases the accident could not be attributed to asphyxia, but directly to the toxic properties of the pental.—*University Medical Magazine*, November, 1893).*

SALICYLIC ACID IN RHEUMATISM.—The external application has been employed for a number of years by Bourget (*Rev. Gener. de Clinique et Therapeutique.—Universety Medical Magazine*, November, 1893). He prescribes the acid as follows:

℞ Salicylic acid............
Lanoline.................
Turpentine....... ...... aa 5 grammes;
Lard..................... 40 grammes.

Apply to the joints twice daily.

The acid is rapidly absorbed, and can be detected in the urine half an hour after the application. According to Bourget, the pain speedily subsides, the temperature falls and resolution is effected in two or three days. The accidents following the internal administration of the drug have not been noted.

THE TREATMENT OF CHLOROSIS.—In the treatment of chlorosis, F. Forchheimer (*Therapeutic Gazette*, November 15, 1893), has used, experimentally, many antiseptics, such as creasote, hydronaphthol, salol, arsenic preparations, bismuth, and tannic acid combinations, and has finally settled down to the routine use of the four former remedies. The hæmoglobin can be increased in every individual on whom he has tried it (eleven cases) by the administration of either salol or hydronaphthol, the latter being, upon the whole, more efficacious in that the rise in hæmoglobin was more rapid. He gives 5 grains of hydronaphthol or salol before meals and the same quantity of hæmogallol immediately after the meal. When these preparations cannot be obtained, large quantities of beef-juice can be substituted, or any of the preparations which contain blood; precaution must be taken to see that they really do contain blood in case the latter are used. In children the author has given up the use of salol for this purpose, and in adults it is used with

* This report sustains the chief conclusions I arrived at in a recent study of the subject, conclusions embodied in my paper entitled "The Actions and Uses of Pental." (See Transactions of the Texas State Medical Association, 1893; also American Medico-Surgical Bulletin, October, 1893.)—D. C.

great caution; in the former it has produced mild carbolic-acid poisoning, and the latter have shown, in several instances, evidences of carbolic acid in the urine. Whether or not salacetol will be a perfect substitute for salol remains to be seen. Second in utility comes the administration of the antiseptic before the meal and some form of iron after the meal; carbonate of iron has given better results than any other preparation the author has used.

LARGE DOSES OF STRYCHNINE IN PULMONARY AND CARDIAC DISEASES.—In an interesting paper, Thomas J. Mays (*The Medical Age*, October 25, 1893), states that his own experiments demonstrate that digitalis acts more on the muscular and less on the nervous structure of the heart than strychnine; that digitalis enhances or increases the irritability of the heart muscle, while strychnine depresses or reduces it; and that the former arrests the heart in systole, while the latter arrests it in diastole. It is believed that the action of these drugs is as dissimilar clinically as it has been shown to be physiologically, and that strychnine is principally indicated in those diseases of the heart which are dependent on a disturbance of innervation, as for example, in simple cardiac weakness and irregularity and intermittency of its pulsations, while digitalis is preferable in cases where there is a want of compensatory power in the heart muscle, as in valvular incompetency. Therefore strychnine should be prescribed when the nerve supply of the heart is enfeebled through auto-intoxication such as is found in the post-paralysis of diphtheria, scarlatina, measles, small-pox, influenza, whooping-cough, and in poisoning from alcohol, lead, mercury, etc. Irregularity and intermittency of the heart's action are frequently benefited by the administration of large doses of strychnine, and more often than not do we find that digitalis is utterly useless in such cases. Sometimes the irregularity will remain, even under the influence of strychnine, but the symptoms which are dependent on, or are a part of this condition, such as pain in the pericardium, orthopnœa, oppression of the chest, will improve or disappear, especially if suitable evacuant remedies are used at the same time. This whole disorder he regards as being probably due to a want of power in the discharge of nerve-force of the heart or rather, perhaps, to a lack of nerve control over the discharge of the muscle-force of the heart. This weakness of nerve-power is not only marked in the heart, but it is also apparent in the lungs, and frequently manifests itself especially in elderly people, in a co-existent œdema of the bases of both lungs. Moreover, there is often found an irregularity or intermittency of the heart's action in severe seizures of asthma; and the author knows of nothing which will remove this accompaniment, as well as the original disease, like strychnine in large doses promptly administered, both hypodermatically and by the mouth. Angina pectoris is another paroxysmal disease in the treatment of which strychnine in large doses stands pre-eminent. Again, digitalis is always regarded as the sovereign remedy in the treatment of valvular diseases of the heart and their sequences, but there comes a period in the life history of every such affection in which digitalis, no matter how much benefit was derived from it before, proves utterly useless. This leads of course to disappointment, and often gives rise to serious suspicions concerning the utility of this important agent. The fault lies, however, not in the drug, but in its improper application. It has done all that could reasonably be expected of it. It stimulated the heart muscle to renewed activity after the valvular rupture occurred. It aided in developing its muscular fibres and restored its former power; but now, for some reason or other, the nervous energy of the patient begins to flag, and the heart-walls commence to relax in spite of the muscular hypertrophy which is present, and digitalis no longer possesses the spurring properties which it once had. The blood dams up in the left ventricle and auricle, the pulmonary circulation becomes impaired, œdema and congestion of the lungs follow, and death is threatened by way of the pulmonary organs. It is at such a time, when digitalis fails to counteract these many incidental complications, that strychnine steps in and shows its superior value as a tonic to the waning nerve energy of the heart and lungs.

